# Methotrexate-associated lymphoproliferative disorder of the thoracic spine in a patient with rheumatoid arthritis receiving methotrexate: a case report

**DOI:** 10.1007/s00256-021-03764-1

**Published:** 2021-03-27

**Authors:** Satoshi Kamio, Ukei Anazawa, Itsuo Watanabe, Aya Sasaki, Ryoma Aoyama

**Affiliations:** 1 Department of Orthopaedic Surgery, Tokyo Dental College Ichikawa General Hospital, Chiba, Japan; 2 Department of Pathology and Laboratory Medicine, Tokyo Dental College Ichikawa General Hospital, Chiba, Japan

**Keywords:** Rheumatoid arthritis, Methotrexate-associated lymphoproliferative disorder, Spinal tumor

## Abstract

Methotrexate-associated lymphoproliferative disorder is recognized as a lymphoma that occurs following methotrexate administration. The lesion of the spine is extremely rare, and only one case of lesion in the lumbar spine has been reported so far. Here, we present a case of methotrexate-associated lymphoproliferative disorder of the thoracic spine in a 54-year-old woman with rheumatoid arthritis. The lesion formed an extra-skeletal tumor mass from lateral to the vertebral body to the paravertebral muscle extending posterior to the epidural space without bone destruction. Magnetic resonance imaging showed low signal intensities on both T1- and T2-weighted images and high signal intensity with short-tau inversion recovery. These radiological findings were similar to those for primary spinal lymphoma. The lesion rapidly paralyzed the patient, forcing her to be treated with posterior spinal decompression. The lesion could not be resected because it adhered to the dura. Following the histopathological diagnosis as methotrexate-associated lymphoproliferative disorder, methotrexate administration was terminated. The remaining mass lesion showed complete regression within 6 months. Methotrexate-associated lymphoproliferative disorder, which could be cured by the discontinuation of methotrexate, should be considered a differential diagnosis in spinal lesion cases showing lymphoma-like appearance with methotrexate treatment to avoid unnecessary treatments.

## Introduction

Methotrexate (MTX), an antimetabolite and immunosuppressive agent, is used in the treatment of rheumatoid arthritis (RA). As opposed to its effectiveness, its serious adverse effects are widely known, and these include liver disorders, interstitial pneumonia, and myelosuppression. Ellman et al. (1991) first reported lymphoma in a patient with RA treated with low-dose MTX [1]. Since then, MTX-associated lymphoproliferative disorder (MTX-LPD) had been recognized as a lymphoma occurring with MTX administration. Some lymphomas are caused by the Epstein-Barr virus (EBV), a well-known oncogenic virus [2]. Reportedly, the frequency of EBV infections in RA patients with lymphoid neoplasms is significantly higher than that in the general population [3]. In patients with RA, MTX administration results in immunosuppression, which activates the EBV, resulting in LPD development [4]. Furthermore, RA is an active inflammatory condition that could cause lymphoma [5]. Therefore, the pathogenesis of this disorder remains unclear.

It has been reported that MTX-LPD remission occurs simply by MTX withdrawal [4, 6]. The frequency of MTX-LPD is similar in the nodal and extranodal regions [4, 7]. Extranodal lesions occur in various locations such as the lung, skin, liver, and oropharyngeal region [5, 8]. However, spinal lesions are extremely rare, and to our knowledge, there is just one report on this type of MTX-LPD lesion [9].

Here, we demonstrate that MTX-LPD in the thoracic spine has a radiologically lymphoma-like appearance. Furthermore, this condition cannot be diagnosed with only radiological and pathological findings, without information of the administration history of MTX. In cases of spinal tumors with a history of MTX administration exhibiting lymphoma-like appearance radiologically, it is important to consider MTX-LPD. This is because MTX-LPD and spinal lymphoma not only show similar images but also require different treatments. The former can be treated by discontinuing MTX, whereas the latter requires radiotherapy, chemotherapy, and, in some cases, surgery.

## Case report

A 54-year-old woman with RA complained of back pain and numbness in the bilateral lower limbs for the past 3 months. She was on MTX (10 mg/week) for approximately 7 years, followed by adalimumab (40 mg per every other week) and iguratimod (50 mg/day) for approximately 3 years. A radiographical assessment by her previous physician revealed a spinal tumor. Therefore, she was referred to our hospital for further investigation and treatment.

The initial examination revealed enhanced bilateral patellar and Achilles reflexes, muscle weakness in the bilateral iliopsoas, a manual muscle testing score of 4, and sensory deficit in the bilateral posterior lower legs. The laboratory investigations revealed the following: white blood cell count, 2900/μL; C-reactive protein level, 0.21 (< 0.3) mg/dL; and anti-cyclic citrullinated peptide antibody titer, 148 (> 4.5) U/mL. Although the antibody titers were high, the C-reactive protein level was within the normal range; this indicated that the progression of RA was well controlled by MTX. Tumor markers, namely, carcinoembryonic antigen level, 0.5 (< 5.0) ng/mL; CA19–9 level, 3.1 (< 37) U/mL; and alpha-fetoprotein level, 5.2 (< 20) ng/mL, were within the normal limits.

**Fig. 1:**
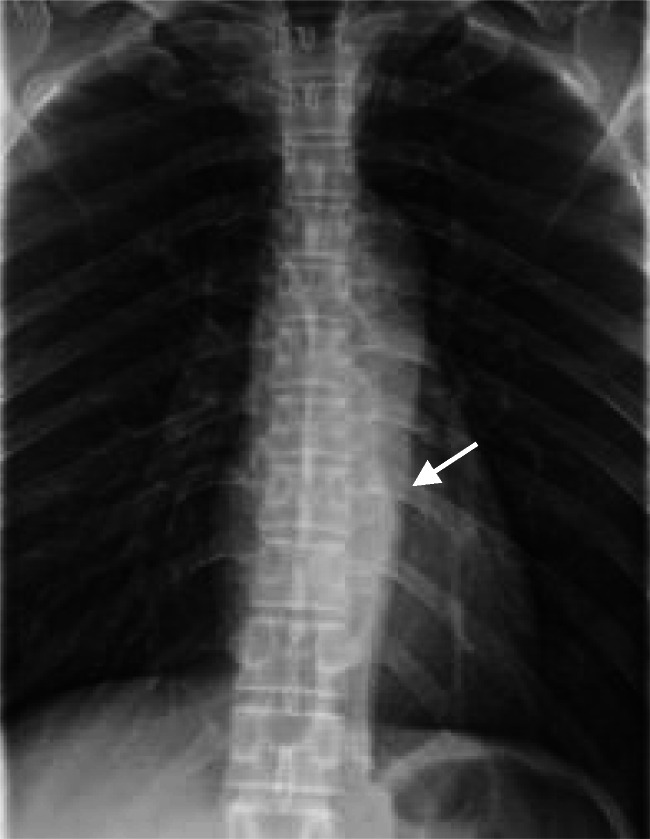
Antero–posterior view of the thoracic spine radiograph. Soft tissue density mass is visualized on the left of Th9–10 (*white arrow*). The mass makes the left transverse process and posterior rib indistinct. This suggests paravertebral lesion

**Fig. 2:**
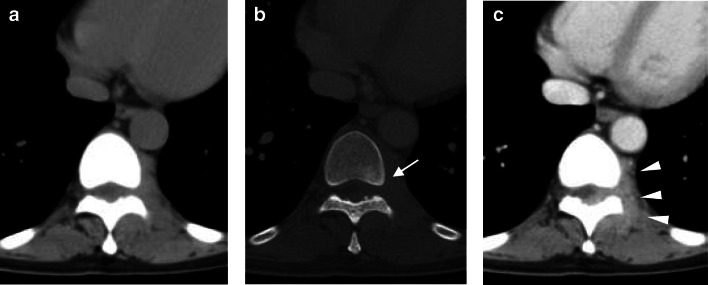
Thoracic spine CT: (**a**) Soft tissue window. (**b**) Bone window. (**c**) Contrast-enhanced CT. Paravertebral mass lesion was observed on the left of the Th10 vertebra. The area adjacent to the tumor-like mass in vertebral body slightly showed bone sclerosis (*white arrow*). The mass lesion and the left side of epidural space and paravertebral muscle were uniformly enhanced. All enhanced area appeared to be connected (*white arrow head*)

Plain radiographs and computed tomography (CT) revealed almost imperceptible bone destruction. Thoracic spine radiograph revealed a soft-tissue mass on the left of Th9–10 (Fig. 1), and a differential diagnosis was considered to be mediastinal tumor. Plain CT revealed a soft-tissue density mass lesion on the left of Th10 (Fig. 2a). The partial sclerotic change of the vertebral body was visualized adjacent to the tumor (Fig. 2b). In contrast-enhanced CT, the mass lesion was well enhanced on the left of epidural space and paravertebral muscle (Fig. 2c). Several lymph nodes swelling were detected in the retrocrural space and mediastinum. Differential diagnoses were considered to be malignant lymphoma and spinal metastasis.

**Fig. 3:**
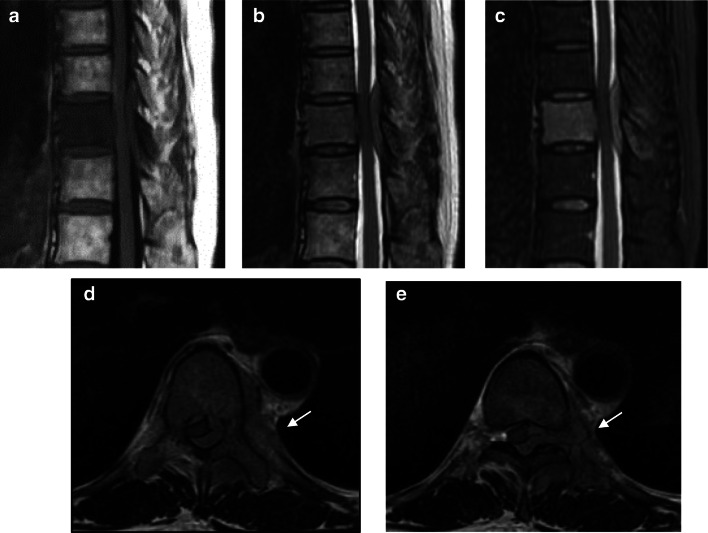
Magnetic resonance imaging of the thoracic spine. (**a–c**) Sagittal image of T1- and T2-weighted images, and short-tau inversion recovery images. (**d, e)** Axial image of T2-weighted images. The lesion on the lateral side of the Th10 vertebral body showed a low signal intensity on the T1- and T2-weighted images and high signal intensity on the short-tau inversion recovery images (*white arrow*). The signal change extended through the pedicle to the posterior elements, including spinous process. Soft-tissue lesion extended to the epidural space, intervertebral foramen, mediastinal area, intercostal space, and paravertebral muscle, showing the same signal intensity as the bone lesion. The epidural lesion compressed the spinal cord. The nodules reminiscent of lymph nodes were revealed on the dorsal side of the aorta. These findings suggest that the vertebral lesion should extend to the epidural space and paravertebral mediastinum and surrounded the muscle without bone destruction

Magnetic resonance imaging (MRI) showed a low signal intensity in the Th10 vertebral body on both T1- and T2-weighted images and a high signal intensity in the short-tau inversion recovery images, and the signal change covered posterior elements through the pedicle. Paravertebral lesion showed the same signal intensity as the bone lesion in each image. This mass lesion extended to the epidural space, mediastinal area, and paravertebral muscle through intervertebral foramen and intercostal space. The epidural mass lesion compressed the spinal cord, causing spinal canal stenosis from the unilateral dorsal site, and this could lead to spinal paralysis (Fig. 3). MRI findings suggested not only malignant lymphoma and spinal metastasis but also myeloma and inflammatory changes as differential diagnoses.

On the basis of these imaging findings, the differential diagnosis included malignant lymphoma, metastatic spinal tumor, hematologic tumor, and inflammatory changes. Blood tests showed no prominent elevation in the inflammatory response or abnormal tumor markers. Furthermore, the patient had no history of cancer, and a CT scan of the whole body showed no malignant tumors in the major organs. Thus, we planned a biopsy.

**Fig. 4:**
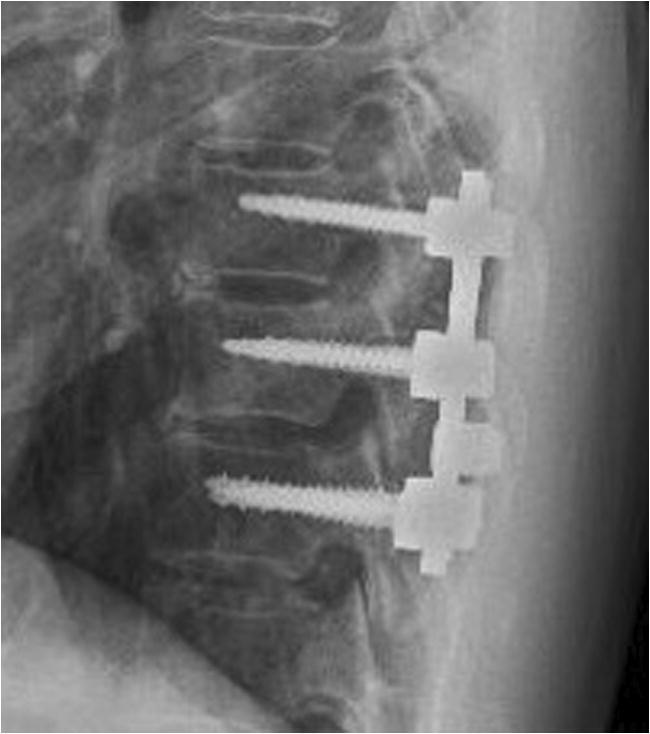
Lateral view of the postoperative plain radiograph. Vertebral decompression and posterolateral fixation of Th9–11 were performed

On day 3 of hospitalization, muscle weakness in the bilateral iliopsoas rapidly progressed, which prevented the patient from walking; it was accompanied by bladder and bowel dysfunctions. Therefore, posterior spinal decompression with laminectomy and posterolateral fixation of the Th9–11 vertebrae were performed as an emergency measure instead of the planned biopsy (Fig. 4). The intraoperative findings confirmed a dark brown neoplastic lesion on the dorsal side of the dura mater at the Th10 vertebral level. The epidural lesion strongly adhered to the dura mater and could not be removed without damaging the dura; therefore, only a pathological specimen was obtained. Consequently, most of the epidural mass lesion was retained.

**Fig. 5:**
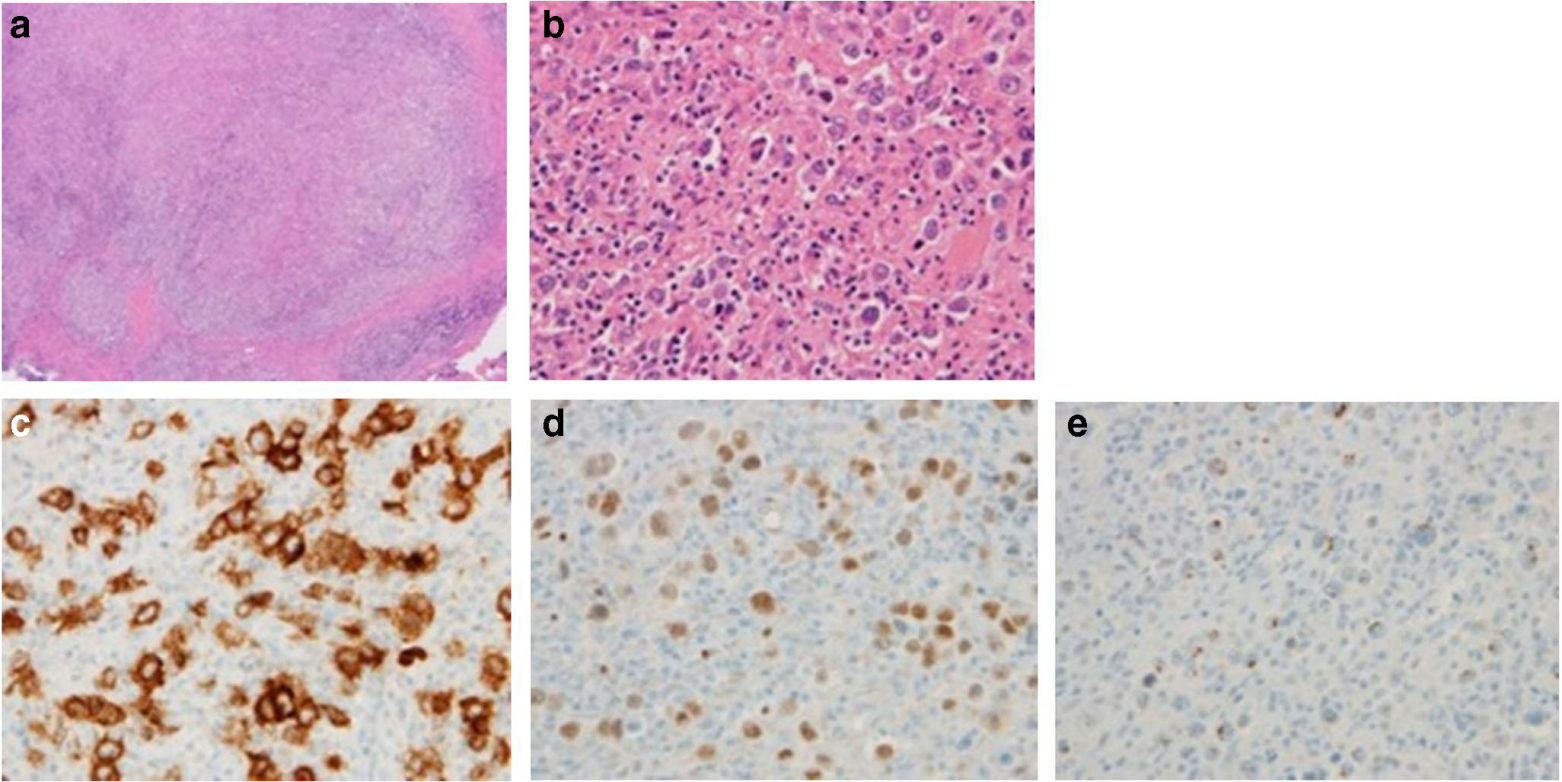
Pathological findings of hematoxylin and eosin staining. (**a**) 40× magnification, **b)** 400× magnification. Pathological findings revealed the proliferation of atypical large round cells accompanied by small lymphocytes in a nodular fashion. Atypical large cells had irregularly shaped nucleus, and a few of them appeared multinucleated. Immunohistochemical staining showed tumor cells positive for (**c**) CD30 and (**d**) Pax5 and (**e**) partially positive for CD15, indicating histological features similar to those of classical Hodgkin lymphoma

The pathological findings revealed the proliferation of atypical large round cells accompanied by small lymphocytes in a nodular fashion. The nuclei in the atypical large cells were irregularly shaped, and a few cells were multinucleated. The immunostaining results were positive for CD30 and Pax5; partially positive for CD4, CD8, CD15, and CD79a; and negative for CD20, pankeratin, and EMA, indicating histological features similar to those of a classical Hodgkin lymphoma (Fig. 5). The results of EBV-encoded RNA in situ hybridization of pathological specimens were negative. Considering both pathological findings and MTX treatment history, this condition was histopathologically diagnosed as MTX-LPD.

**Fig. 6:**
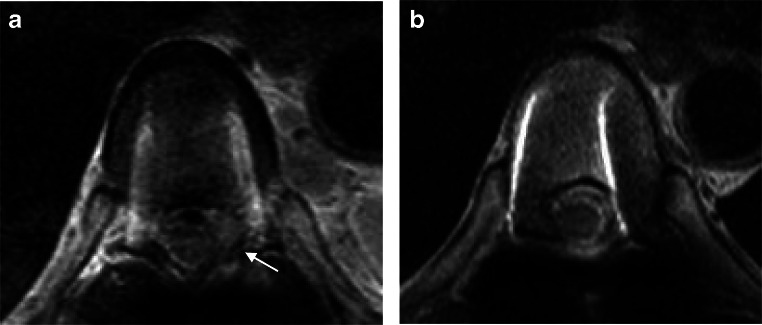
Postoperative T2-weighted magnetic resonance images (axial view). (**a**) One month after surgery. The spinal cord appeared sufficiently decompressed. Partially resected tumor in epidural space and the remaining tumor on the lateral side (*white arrows*) were confirmed. (**b**) Six months after surgery. Both epidural and lateral vertebral masses were not observed

An improvement in bilateral iliopsoas muscle weakness and bladder rectal dysfunction was observed postoperatively, and the patient could resume walking. Postoperative investigation demonstrated a high level of anti-EBV capsid antigen-IgG, at 320 times the normal level (normal level, < 10-fold), suggesting a history of infection. After being diagnosed with MTX-LPD, treatment with MTX was terminated. The symptoms of RA did not worsen, and prednisolone 5 mg/day was administered as an alternative to MTX from 1 week after surgery. Three weeks after MTX withdrawal, the MRI findings revealed a partially resected epidural tumor mass with decompressed spinal cord. Moreover, a remnant tumor was noted on the lateral side of the vertebral body. The disappearance of both tumors was confirmed within 6 months after surgery (Fig. 6). Eighteen months after the surgery, the patient could walk independently and did not show tumor recurrence.

## Discussion

For spinal tumors with progressive symptoms, an accurate diagnosis is essential for determining the appropriate treatment, because chemo- or radio-sensitive tumors can be cured without spinal surgery, which has a complication rate of 21.7–34% [10–12]. However, most of these tumors demonstrate only atypical radiological appearances, and they require biopsy specimens for a definitive diagnosis [13]. Therefore, for tumors that cause rapid paralysis, emergency spinal surgery is performed to obtain the pathological specimen and decompress the spinal cord.

MTX-LPD can show remission after MTX withdrawal without chemotherapy and radiotherapy, and it has a characteristic medical history and condition. Reports of this disorder are more from Japan than from other countries, but it is unclear whether the reason is local perception or racial differences [6]. The World Health Organization classification of hematopoietic and lymphoid tumors classifies MTX-LPD as the “other iatrogenic subgroup of immunodeficiency-associated lymphoproliferative disorders” [14].

Histopathologically, MTX-LPD has several subtypes. MTX-LPD in the present case was similar to classical Hodgkin lymphoma and comprised large round cells and small lymphocytes with multiple nuclear features. The most common subtype of MTX-LPD is diffuse large B cell lymphoma, which accounts for nearly half of all cases. The second most common subtype is Hodgkin lymphoma, which has been reported in approximately 10–20% of patients [4, 6, 15, 16]. Furthermore, complex phenotypes, including atypical peripheral T cell lymphoma, have also been reported [17].

The clinical characteristics of MTX-LPD include a history of MTX treatment for approximately 30 months or more, previous EBV infection, and the possibility of remission upon MTX discontinuation [18]. The current case demonstrated all features of MTX-LPD: a history of MTX treatment (10 mg/week) for 7 years, a high anti-EBV capsid antigen-IgG level, and complete remission within 6 months post MTX discontinuation.

Kameda et al. surveyed 5753 patients with RA in Japan and compared 125 patients without LPD receiving MTX and 28 patients with MTX-LPD. They found that MTX at a concentration of 8 mg/week or more is an independent risk factor for the development of LPD [7]. Furthermore, previous EBV infection may initiate LPD. Hoshida et al. reported that RA patients with LPD have a significantly higher EBV infection rate than those with sporadic LPD [4]. An immunodeficient condition with MTX treatment is considered a basis for the onset of LPD caused by EBV. Furthermore, RA itself could be a cause of lymphoma because of the associated inflammatory condition [5]. It is apparent that an accidental LPD occurred in our patient treated with MTX. Thus, the pathogenesis of this condition is still unknown, including its relationship with MTX.

The primary treatment option is the discontinuation of MTX. Previous studies have reported that almost half of the MTX-LPD cases show regression with MTX withdrawal alone [4, 17]. A few studies have indicated that EBV-infected patients are likely to show remission [15, 17]. In a few studies, some patients showed a temporary disease regression followed by recurrence, whereas others only showed rapid disease progression post-MTX withdrawal [6, 17]. For these non-responders to MTX withdrawal, conventional chemotherapy for lymphoma should be considered [4]. Rizzi et al. reviewed published data of 26 patients and found that complete remission mostly occurs within 4 weeks after the discontinuation of MTX and other immunosuppressive agents [19]. In contrast, Inui et al. reported the maximum tumor shrinkage only after 8 weeks of MTX termination in 13 of 15 patients [20]. Therefore, follow-up after 8 weeks or more should be favorable to evaluate the response of the tumor.

To the best of our knowledge, only one case of MTX-LPD arising from the spine has been reported, and it demonstrated an epidural tumor mass and complete remission after MTX discontinuation, as in the current case [9]. The predominant site of this tumor was the nodal region, and it occupied half of the primary site [4, 6]. However, the location of extralymphatic lesions varied, including the lungs, skin, liver, and oropharynx. The occurrence of a bone lesion is particularly rare. Kameda et al. demonstrated a frequency of one out of 28 patients (3.5%) [7]. Moreover, multiple bone lesions and femoral pathological fractures have been reported [21].

In terms of radiographic appearance, the previous case of spinal MTX-LPD showed findings similar to those observed in the present case, with almost imperceptible bone destruction and low signal intensity on T1- and T2-weighted images of both spinal and epidural lesions [9]. In this case, the tumor extended to the surrounding soft tissue with lymph node swelling, and it had to be differentiated from bone metastasis and infection. However, she did not have a history of cancer, abnormal tumor marker levels, no abnormal lesion in major organs, and no prominent elevation in inflammatory response. In spinal primary lymphoma, most of the bone lesions demonstrated either a permeative pattern, including almost imperceptible bone destruction, or moth-eaten pattern, showing multiple lytic lesions [22]. In the MRI, they demonstrated a low signal intensity on the T1-weighted images and variable signal intensity on the T2-weighted images, and the location of epidural mass in them was often dorsal rather than ventral [23]. The radiological appearance of spinal MTX-LPDs in the previous case and present case included the features of primary spinal lymphoma.

In conclusion, the MRI of the spinal tumor of a patient with RA receiving MTX demonstrated a primary spinal lymphoma and the histological findings demonstrated classical Hodgkin lymphoma. Only radiological findings and histopathological findings could not distinguish spinal lymphoma from spinal MTX-LPD. In spinal lymphoma-like tumor, the medical history is important to distinguish spinal MTX-LPD from spinal lymphoma. Clinically, MTX-LPD, which could be cured by MTX withdrawal alone, should be considered, when lymphoma-like spinal tumor develops in RA patients with a history of MTX. Further research is required to identify the radiological features and the treatments of this spinal disorder because this type of spinal tumor is very rare.
